# Association Between the Uric Acid-to-Creatinine Ratio and Elevated Parathyroid Hormone Levels in Adults Without Known Parathyroid Disease

**DOI:** 10.3390/jcm15135285

**Published:** 2026-07-07

**Authors:** Orhan Özdemir, Ahmet Ziya Şahin, Eray Çetin, Zeynel Abidin Sayiner

**Affiliations:** 1 Department of Nephrology, Sanko University Faculty of Medicine, Gaziantep 27470, Türkiye; drorhanozdemir@hotmail.com; 2 Department of Nephrology, Health Sciences University Adana City Hospital, Adana 01370, Türkiye; 3 Department of Internal Medicine, Sanko University Faculty of Medicine, Gaziantep 27470, Türkiye; fevzieraycetin2@gmail.com; 4 Department of Endocrinology and Metabolism, Sanko University Faculty of Medicine, Gaziantep 27470, Türkiye; zeynelasayiner@hotmail.com

**Keywords:** parathyroid, uric acid-to-creatinine ratio, vitamin D, mineral metabolism

## Abstract

**Background:** Parathyroid hormone (PTH) is mainly regulated by calcium, vitamin D, and renal function, but metabolic factors may also influence its levels. The uric acid/creatinine (UA/Cr) ratio reflects uric acid metabolism and renal handling. This study evaluated its association with elevated PTH in adults without known parathyroid disease. **Methods:** This retrospective cross-sectional study included 244 adults undergoing routine biochemical evaluation. The uric acid-to-creatinine ratio (UA/Cr) was calculated. Participants were categorized according to PTH levels (≤65 pg/mL vs. >65 pg/mL). Logistic regression analyses were performed to identify factors associated with elevated PTH levels. **Results:** A total of 244 individuals (mean age 40.6 ± 11.7 years; 70.9% female) were included in the analysis. Participants with elevated PTH levels had significantly higher UA/Cr ratios compared with those with normal PTH levels (8.93 ± 1.82 vs. 7.47 ± 1.79, *p* = 0.031). In univariate logistic regression analysis, UA/Cr was associated with elevated PTH (OR 1.15, 95% CI 1.01–1.32, *p* = 0.043). In multivariable logistic regression analysis adjusting for vitamin D, magnesium, phosphorus, corrected calcium, age, sex, body mass index, and the estimated glomerular filtration rate, the UA/Cr ratio remained significantly associated with elevated PTH levels (OR 1.41, 95% CI 1.036–1.66, *p* = 0.024). **Conclusions:** The UA/Cr ratio was associated with elevated PTH levels in adults without known parathyroid disease, independently of vitamin D status, mineral metabolism parameters, and renal function. These findings suggest that the UA/Cr ratio may help characterize a broader metabolic context associated with elevated PTH levels, rather than serving as a diagnostic marker. Further prospective studies are needed to clarify the clinical relevance and direction of this association.

## 1. Introduction

Parathyroid hormone (PTH) plays a central role in the regulation of calcium phosphate homeostasis and bone metabolism. Physiologically, PTH secretion is tightly regulated by extracellular calcium levels through the calcium sensing receptor, while vitamin D status and renal function represent additional important modulators of PTH regulation [1]. Classical models of secondary hyperparathyroidism primarily emphasize disturbances in mineral metabolism, particularly vitamin D deficiency, hypocalcemia, and chronic kidney disease, as key determinants of elevated PTH levels [2]. However, increasing evidence suggests that PTH regulation may also be influenced by metabolic and systemic factors beyond the classic calcium–phosphate axis. Several epidemiological and clinical studies have reported associations between PTH concentrations and metabolic disturbances including obesity, insulin resistance, oxidative stress, and hyperuricemia [3,4]. These findings have led to the hypothesis that elevated PTH may reflect a broader metabolic milieu rather than solely alterations in mineral metabolism.

Uric acid, the final product of purine metabolism in humans, has emerged as an important marker of metabolic stress and oxidative imbalance. Elevated uric acid levels have been associated with cardiometabolic disorders, renal dysfunction, and systemic inflammatory states, and recent large-scale prospective evidence has further supported the comorbidity burden associated with hyperuricemia and gout [5]. However, serum uric acid levels are influenced not only by purine metabolism but also by renal excretion, which accounts for nearly two-thirds of total uric acid elimination. Because renal function directly affects circulating uric acid concentrations, isolated serum uric acid measurements may not fully reflect underlying metabolic alterations. Adjustment for creatinine, a widely used surrogate of renal filtration and muscle-related metabolic turnover, may provide a more integrated assessment of uric acid metabolism. In this context, the uric acid-to-creatinine ratio (UA/Cr) has been proposed as a composite metabolic index that reflects both uric acid production and renal handling. By accounting for interindividual variability in renal function, the UA/Cr ratio may therefore better capture metabolic stress and systemic metabolic disturbances compared with serum uric acid alone [6,7]. Although previous studies have explored the relationship between uric acid and parathyroid hormone, results have been inconsistent and the underlying mechanisms remain incompletely understood. Moreover, the potential role of uric acid derived metabolic indices, such as the uric acid-to-creatinine ratio, in relation to elevated PTH levels has not been adequately investigated. Understanding whether metabolic parameters are associated with PTH levels independently of classical mineral metabolism markers could provide new insights into the broader physiological determinants of parathyroid hormone regulation.

This study aimed to determine whether the uric acid-to-creatinine ratio is associated with elevated PTH levels in individuals without known parathyroid disease beyond determinants of parathyroid hormone regulation, including calcium, phosphorus, magnesium, vitamin D status, and renal function.

## 2. Materials and Methods

This retrospective cross-sectional study included 244 adult outpatients who underwent routine biochemical evaluation at Sanko University between 1 January 2025 and 31 December 2025. Adult patients aged ≥ 18 years with available measurements of serum parathyroid hormone, uric acid, creatinine, calcium, phosphorus, 25-hydroxyvitamin D, and magnesium were included in the analysis.

Exclusion criteria included primary hyperparathyroidism, known parathyroid disease, prior parathyroid surgery, hypercalcemia suggestive of primary hyperparathyroidism (when clinically indicated, neck ultrasonography), known kidney disease or estimated glomerular filtration rate < 60 mL/min/1.73 m^2^, active malignancy, chronic inflammatory or autoimmune diseases, hypertension, acute infection at the time of evaluation, and chronic liver disease. Patients with metabolic bone diseases, including osteomalacia, Paget’s disease, or known osteoporosis requiring active treatment, were also excluded [8]. Patients receiving medications known to influence calcium phosphate metabolism, such as calcimimetics, vitamin D supplements, or high-dose calcium supplementation, were also excluded. When available, medical records were reviewed for information on recent consumption of calcium-rich foods, including dairy products, during the three days before blood sampling, to account for the potential influence of short-term dietary calcium intake on parathyroid hormone levels.

### 2.1. Laboratory Measurements

Blood samples were obtained after overnight fasting and analyzed in the hospital’s central laboratory using standardized automated analyzers. The biochemical parameters evaluated in the present study included serum parathyroid hormone (PTH), 25-hydroxyvitamin D [25(OH)D], uric acid, creatinine, corrected calcium, phosphorus, magnesium, high-density lipoprotein (HDL) cholesterol, low-density lipoprotein (LDL) cholesterol, triglycerides, C-reactive protein (CRP), and urine albumin-to-creatinine ratio. Hematological parameters included hemoglobin, white blood cell count, platelet count, neutrophil count, and lymphocyte count.

Serum PTH, 25(OH)D, uric acid, creatinine, calcium, phosphorus, and magnesium levels were measured using the Abbott Alinity Series analyzer system (Abbott Laboratories, Abbott Park, IL, USA) in accordance with the manufacturer’s instructions. Serum PTH levels were measured using a chemiluminescent microparticle immunoassay (CMIA) method and reported as pg/mL; the reference range was 15–65 pg/mL. Serum 25(OH)D levels were also measured using the CMIA method and reported as ng/mL; the reference range for vitamin D sufficiency was 30–100 ng/mL.

Serum uric acid, creatinine, calcium, phosphorus, and magnesium levels were analyzed on the Abbott Alinity c clinical chemistry analyzer using spectrophotometric methods. Uric acid was measured using the uricase-POD enzymatic colorimetric method, with reference ranges of 3.5–7.2 mg/dL for men and 2.6–6.0 mg/dL for women. Creatinine was measured using an enzymatic method, with a reference range of 0.6–1.2 mg/dL. Calcium was measured using the Arsenazo III colorimetric method, with a reference range of 8.6–10.2 mg/dL. Phosphorus was measured using the phosphomolybdate UV method, with a reference range of 2.5–4.5 mg/dL. Magnesium was measured using the xylidyl blue colorimetric method, with a reference range of 1.7–2.4 mg/dL.

Serum HDL cholesterol, LDL cholesterol, triglyceride, and CRP levels were analyzed using a Roche Diagnostics Cobas c702 analyzer (Roche Diagnostics, Mannheim, Germany). CRP was measured using an immunoturbidimetric method, with a reference range of 0–5 mg/L. HDL cholesterol was measured using an enzymatic colorimetric method, with reference values of >40 mg/dL for men and >50 mg/dL for women. LDL cholesterol was measured using a direct homogeneous method, with a reference value of <100 mg/dL. Triglyceride levels were measured using an enzymatic colorimetric method, with a reference value of <150 mg/dL.

Complete blood count analyses were performed using EDTA-anticoagulated venous blood samples on a Sysmex XN Series automated hematology analyzer (Sysmex Corporation, Kobe, Japan). Leukocyte differentiation, neutrophil counts, and lymphocyte counts were determined using fluorescence flow cytometry. Platelet counts were measured using the direct current sheath flow impedance method with hydrodynamic focusing, and hemoglobin levels were measured using the sodium lauryl sulfate hemoglobin photometric method. The accepted reference ranges were 4.0–10.0 × 10^3^/µL for white blood cell count, 1.8–7.5 × 10^3^/µL for neutrophil count, 1.0–4.0 × 10^3^/µL for lymphocyte count, 150–400 × 10^3^/µL for platelet count, 12.0–17.5 g/dL for hemoglobin.

Urinary albumin and creatinine concentrations were measured from the same spot urine sample. Urinary albumin was measured using an immunoturbidimetric method, while urinary creatinine was measured using an enzymatic method on an Abbott ARCHITECT/Alinity automated clinical chemistry analyzer. The urinary albumin-to-creatinine ratio was calculated by dividing urinary albumin concentration by urinary creatinine concentration and was expressed as mg/g creatinine.

The estimated glomerular filtration rate was calculated using the CKD-EPI equation. Corrected calcium was calculated according to serum calcium and albumin levels. The uric acid-to-creatinine ratio (UA/Cr) was calculated by dividing serum uric acid levels by serum creatinine levels, and the uric acid-to-HDL cholesterol ratio was calculated by dividing serum uric acid levels by HDL cholesterol levels. The neutrophil-to-lymphocyte ratio was calculated by dividing the absolute neutrophil count by the absolute lymphocyte count.

Elevated PTH levels were defined as PTH > 65 pg/mL because 65 pg/mL represents the upper limit of the reference range for the intact PTH assay used in the hospital’s central laboratory. This threshold was therefore based on the laboratory-defined reference range rather than a study-derived statistical cut-off. Patients were categorized into two groups according to PTH levels: normal PTH (≤65 pg/mL) and elevated PTH (>65 pg/mL). Vitamin D deficiency was defined as serum 25(OH)D levels below 20 ng/mL.

All internal quality control procedures were performed daily, and analyzer calibrations were carried out according to the manufacturers’ recommendations.

### 2.2. Statistical Analysis

Statistical analyses were performed using IBM SPSS Statistics for Windows, version 26.0. Continuous variables were expressed as mean ± standard deviation (SD) or median (interquartile range) depending on data distribution, and categorical variables were presented as numbers and percentages. Comparisons between groups according to PTH status were performed using Student’s *t*-test or the Mann–Whitney U test for continuous variables and the chi-square test for categorical variables. The normality of continuous variables was assessed using visual inspection of histograms and the Kolmogorov–Smirnov test. Logistic regression analyses were performed to evaluate factors associated with elevated PTH levels. Variables included in the multivariate model were the UA/Cr ratio, 25-hydroxyvitamin D, magnesium, phosphorus, corrected calcium, age, sex, BMI, and eGFR. Given the imbalance in sex distribution within the study population, sex was included as a covariate in the logistic regression analysis to account for its potential confounding effect on the association between the UA/Cr ratio and elevated PTH levels. A post hoc power analysis was performed using G*Power software, version 3.1.9.7, to assess whether the final sample size was sufficient for the primary comparison of UA/Cr ratios between the normal PTH and elevated PTH groups. The analysis was based on a two-tailed independent samples *t*-test, an alpha error probability of 0.05, the observed effect size for the difference in UA/Cr ratios between groups (Cohen’s d = 0.81), and the actual group sizes (normal PTH group, *n* = 137; elevated PTH group, *n* = 107). The calculated statistical power was >0.99, indicating that the final sample size was sufficient to detect the observed between-group difference in UA/Cr ratios. As an additional sensitivity analysis, PTH was also evaluated as a continuous dependent variable. Because PTH showed a skewed distribution, log-transformed PTH was used in linear regression models. The unadjusted model included only the UA/Cr ratio, whereas the adjusted model included age, sex, BMI, eGFR, corrected calcium, phosphorus, magnesium, and 25-hydroxyvitamin D. A *p* value < 0.05 was considered statistically significant.

## 3. Results

A total of 244 adults were included in the analysis. The mean age of the study population was 40.75 ± 11.79 years, and the mean body mass index (BMI) was 25.83 ± 3.41 kg/m^2^. The mean estimated glomerular filtration rate (eGFR) was 106.89 ± 16.79 mL/min/1.73 m^2^. The mean serum uric acid level was 5.59 ± 1.49 mg/dL, serum creatinine 0.74 ± 0.15 mg/dL (0.6–1.2 mg/dL), and the calculated uric acid-to-creatinine ratio (UA/Cr) was 8.11 ± 1.82. The mean corrected calcium level was 9.29 ± 0.47 mg/dL (8.6–10.2 mg/dL), phosphorus 3.34 ± 0.55 mg/dL (2.5–4.5 mg/dL), magnesium 1.97 ± 0.17 mg/dL (1.7–2.4 mg/dL), and C-reactive protein 4.19 ± 4.65 mg/L (<5 mg/L). Hematological inflammatory markers demonstrated a mean neutrophil-to-lymphocyte ratio (NLR) of 2.05 ± 1.20. Detailed baseline clinical and biochemical characteristics are presented in Table 1.

Participants were categorized according to parathyroid hormone levels as PTH ≤ 65 pg/mL (*n* = 137) and PTH > 65 pg/mL (*n* = 107). The distribution of sex and magnesium categories did not differ significantly between the two groups (*p* = 0.784 and *p* = 0.802, respectively). The uric acid-to-creatinine ratio was significantly higher in individuals with PTH > 65 pg/mL compared with those with PTH ≤ 65 pg/mL (8.93 ± 1.82 vs. 7.47 ± 1.79, *p* = 0.031). No significant differences were observed between the groups for age (*p* = 0.730), corrected calcium (*p* = 0.906), serum uric acid (*p* = 0.099), eGFR (*p* = 0.795), phosphorus (*p* = 0.162), or uric acid/HDL ratio (*p* = 0.470). In addition, BMI (*p* = 0.620), NLR (*p* = 0.447), and urine albumin/creatinine ratio (*p* = 0.446) were similar between the PTH groups.

Participants were also stratified according to vitamin D status as vitamin D < 20 ng/mL (*n* = 60) and vitamin D ≥ 20 ng/mL (*n* = 184). The distribution of sex and magnesium categories did not differ significantly between vitamin D groups (*p* = 0.445 and *p* = 0.656). Most biochemical parameters, including the UA/Cr ratio (*p* = 0.069), serum uric acid (*p* = 0.267), corrected calcium (*p* = 0.297), phosphorus (*p* = 0.491), and eGFR (*p* = 0.088), were also similar between the groups. BMI (*p* = 0.305) and the urine albumin/creatinine ratio (*p* = 0.636) were not significantly different between the vitamin D groups (Table 2).

Logistic regression analyses were performed to evaluate factors associated with elevated PTH levels. In the univariate model, the uric acid–creatinine ratio showed a significant association with elevated PTH levels (OR 1.15, 95% CI 1.01–1.32, *p* = 0.043). In multivariable logistic regression analysis including vitamin D and other clinical variables, the uric acid-to-creatinine ratio remained independently associated with elevated PTH levels (OR 1.41, 95% CI 1.036–1.66, *p* = 0.024) (Table 3).

As a sensitivity analysis, PTH was also analyzed as a continuous dependent variable. Because PTH showed a skewed distribution, log-transformed PTH was used in linear regression analysis. In the unadjusted model, the uric acid-to-creatinine ratio was significantly and positively associated with log-transformed PTH levels (B = 0.038, 95% CI: 0.009 to 0.067, *p* = 0.010). This association remained significant after adjustment for age, sex, BMI, eGFR, corrected calcium, phosphorus, magnesium, and 25-hydroxyvitamin D (B = 0.033, 95% CI: 0.005 to 0.061, *p* = 0.020). The fully adjusted model explained 19.1% of the variance in log-transformed PTH levels (R^2^ = 0.191, adjusted R^2^ = 0.160, *p* < 0.001) (Table 4).

## 4. Discussion

High PTH levels have been primarily linked to disorders in mineral metabolism, particularly vitamin D deficiency, hypocalcemia, and renal dysfunction [9]. However, increasing evidence suggests that PTH levels may also be influenced by broader metabolic processes. Numerous population-based studies have reported associations between PTH concentrations and metabolic abnormalities such as obesity, insulin resistance, and cardiovascular risk factors [10,11]. In the present study, the uric acid-to-creatinine ratio (UA/Cr) was significantly higher in individuals with elevated PTH levels and remained independently associated with elevated PTH after adjustments for vitamin D status, mineral metabolism parameters, renal function, age, sex, and BMI. In addition, the sensitivity analysis using log-transformed continuous PTH supported the primary findings. The UA/Cr ratio remained significantly associated with PTH when PTH was modeled as a continuous biological variable rather than a dichotomous outcome. This finding reduces the possibility that the observed association was solely driven by the selected clinical cutoff of 65 pg/mL and suggests that higher UA/Cr ratios may be related to higher PTH levels across the study population.

Uric acid is the product of purine metabolism and is increasingly recognized as a marker of metabolic stress and oxidative imbalance [12,13]. Experimental studies have shown that uric acid may contribute to oxidative stress, endothelial dysfunction, and inflammation, which in turn may affect hormonal regulatory systems [6,14]. The UA/Cr ratio may provide a more integrated metabolic indicator than serum uric acid alone because creatinine partially accounts for renal filtration and interindividual differences in uric acid excretion. Therefore, the observed association between the UA/Cr ratio and elevated PTH levels may suggest that metabolic alterations related to uric acid production or renal urate handling could be linked to variability in PTH regulation in individuals without known parathyroid disease.

Previous studies examining the relationship between uric acid and PTH have reported inconsistent findings, with some studies demonstrating positive correlations between serum uric acid levels and PTH concentrations, whereas others have found no significant association [13,15]. Several pathophysiological mechanisms may explain the observed association between elevated PTH levels and the UA/Cr ratio. Elevated PTH may influence renal urate handling through effects on proximal tubular urate transporters, renal tubular function, and mineral metabolism, thereby contributing to reduced uric acid excretion or altered serum uric acid concentrations [16,17]. Conversely, increased uric acid burden may affect PTH regulation by inhibiting renal 1-α-hydroxylase activity, reducing the synthesis of active vitamin D, and subsequently stimulating PTH secretion [16,17]. In addition, hyperuricemia has been associated with oxidative stress, endothelial dysfunction, and low-grade inflammatory signaling pathways, which may indirectly influence hormonal regulatory systems, including vitamin D metabolism and parathyroid hormone secretion [18]. These mechanisms may also help explain why previous findings have been inconsistent, as the relationship between uric acid metabolism and PTH regulation may vary according to renal function, vitamin D status, calcium–phosphate balance, inflammatory activity, and metabolic comorbidities. However, because the present study has a retrospective cross-sectional design, these mechanisms should be interpreted as plausible biological explanations for the observed association rather than evidence of causality.

Studies in the literature report a relationship between hyperuricemia and higher PTH levels; however, most studies have focused solely on serum uric acid levels, and compound indices that more comprehensively reflect uric acid metabolism have not been sufficiently evaluated [6]. In this study, the uric acid-to-creatinine ratio was higher in individuals with elevated PTH levels compared with those with normal PTH concentrations. Furthermore, UA/Cr levels did not significantly differ between vitamin D deficiency groups. These findings suggest that the observed relationship between UA/Cr and PTH levels may not be explained solely by vitamin D deficiency or classical disturbances of mineral metabolism. However, given the cross-sectional design of the study, the direction of this relationship cannot be determined, and no causal inference can be made. From a clinical perspective, the UA/Cr ratio should not be interpreted as a diagnostic marker for elevated PTH levels but rather as a potential metabolic indicator associated with PTH variability. The findings suggest that elevated PTH concentrations in individuals without known parathyroid disease may occur within a broader metabolic context that includes alterations in uric acid metabolism. Further studies are needed to clarify whether the UA/Cr ratio reflects underlying metabolic stress pathways that influence PTH regulation.

This study has certain limitations. The cross-sectional study design does not allow for establishing causality between the uric acid-to-creatinine ratio and elevated parathyroid hormone levels. Furthermore, biochemical parameters, including PTH and vitamin D levels, are based on a single measurement and may not fully reflect long-term metabolic status. The study was conducted at a single clinical center; therefore, the generalizability of the findings may be limited. Furthermore, additional metabolic or hormonal factors not evaluated in this study, such as fibroblast growth factor-23 (FGF-23) and 1,25-dihydroxyvitamin D, may also contribute to the variability in parathyroid hormone levels. Another limitation is the imbalance in sex distribution, as nearly 80% of the study population consisted of female participants. Although sex was included as a covariate in the regression analysis, this imbalance may still limit the generalizability of the findings, particularly to male populations.

In conclusion, elevated parathyroid hormone levels in individuals without known parathyroid disease were associated with higher uric acid-to-creatinine ratios. These findings suggest that the UA/Cr ratio may be a metabolic correlate of elevated PTH levels beyond established regulators of mineral metabolism.

## Figures and Tables

**Table 1:** Baseline clinical and biochemical characteristics of the study population.

Variables	Min	Max	Mean	SD
Age (years)	19	72	40.75	11.789
BMI (18.5–24.9 kg/m^2^)	18.50	38.60	25.83	3.418
eGFR (>60 mL/min/1.73 m^2^)	60.89	161.21	106.89	16.798
Creatinine (0.6–1.2 mg/dL)	0.49	1.34	0.74	0.147
Uric Acid (3.5–7.2 mg/dL)	2.30	9.40	5.59	1.487
HDL (>40 mg/dL M, >50 mg/dL F)	28	91	51.52	12.227
Uric Acid/HDL	0.04	0.34	0.12	0.050
Uric Acid/Creatinine	3.18	14.67	8.11	1.819
Triglycerides (<150 mg/dL)	22.00	367.00	117.85	61.682
LDL (<100 mg/dL)	11.60	198.80	121.09	34.078
PTH (pg/mL)	11	268.2	70.58	35.51
25-hydroxyvitamin D (ng/mL)	5.00	77.6	27.46	12.22
CRP (<5 mg/L)	1.00	24.70	4.19	4.652
Corrected Ca (8.6–10.2 mg/dL)	8.10	10.40	9.29	0.467
Phosphorus (2.5–4.5 mg/dL)	1.30	4.90	3.34	0.551
Magnesium (1.7–2.4 mg/dL)	1.20	3.20	1.97	0.175
NLR (1.0–3.0)	0.59	12.35	2.05	1.198
Hemoglobin (HGB) (12.0–17.5 g/dL)	9.80	17.70	13.99	1.415
WBC (4.0–10.0 ×10^3^/µL)	3.34	15.54	7.14	2.091
PLT (150–400 ×10^3^/µL)	103.00	457.00	269.09	58.469
UrineALB/CRE (mg/g) (<30 mg/g)	4.00	241.00	29.62	40.628

**Table 2:** Comparison of clinical and biochemical parameters according to PTH status and vitamin D status.

	PTH ≤ 65 pg/mL (*n* = 137)	PTH > 65 pg/mL (*n* = 107)		Vit D < 20 ng/mL (*n* = 60)	Vit D ≥ 20 ng/mL (*n* = 184)	*p*
Variables	*n* (%)	*n* (%)	*p*	*n* (%)	*n* (%)	*p*
Sex (Female)	102 (56.7)	78 (43.3)	0.784	42 (23.3)	138 (76.7)	0.445
Sex (Male)	35 (54.7)	29 (45.3)		18 (28.1)	46 (71.9)	
Magnesium (Low)	41 (59.4)	28 (40.6)		16 (23.2)	53 (76.8)	
Magnesium (Normal)	63 (55.3)	51 (44.7)	0.802	31 (27.2)	83 (72.8)	0.656
Magnesium (High)	33 (54.1)	28 (45.9)		13 (21.3)	48 (78.7)	
	Mean ± SD	Mean ± SD	*p*	Mean ± SD	Mean ± SD	*p*
Age (years)	40.99 ± 11.880	40.46 ± 11.720	0.730	38.83 ± 11.551	41.38 ± 11.829	0.146
Corrected Ca (8.6–10.2 mg/dL)	9.30 ± 0.466	9.29 ± 0.470	0.906	9.24 ± 0.441	9.31 ± 0.475	0.297
Uric Acid (3.5–7.2 mg/dL)	5.45 ± 1.503	5.77 ± 1.453	0.099	5.78 ± 1.456	5.53 ± 1.496	0.267
Uric Acid/Creatinine (ratio)	7.47 ± 1.797	8.93 ± 1.82	0.031	8.04 ± 1.813	7.55 ± 1.810	0.069
eGFR (mL/min/1.73 m^2^)	107.14 ± 16.490	106.57 ± 17.258	0.795	110.10 ± 14.775	105.84 ± 17.316	0.088
Phosphorus (2.5–4.5 mg/dL)	3.29 ± 0.537	3.39 ± 0.567	0.162	3.38 ± 0.514	3.32 ± 0.563	0.491
	Mean rank	Mean rank	*p*	Mean rank	Mean rank	*p*
BMI (kg/m^2^)	124.48	119.97	0.620	130.61	119.86	0.305
UrineALB/CRE (mg/g) (<30 mg/g)	125.54	118.61	0.446	118.76	123.72	0.636

**Table 3:** Logistic regression analysis of factors associated with elevated PTH levels.

Model	Independent Variable	B	OR (95% CI)	*p* Value
Univariate	Uric acid/creatinine ratio	0.14	1.15 (1.01–1.32)	0.043
Multivariable	Uric acid/creatinine ratio	0.34	1.41 (1.036–1.66)	0.024
Multivariable	Vitamin D	−0.06	0.94 (0.91–0.96)	0.001
Multivariable	Magnesium	0.18	1.19 (0.67–2.14)	0.534
Multivariable	Phosphorus	0.31	1.37 (0.82–2.27)	0.220
Multivariable	Corrected calcium	0.01	1.01 (0.54–1.89)	0.952
Multivariable	Age	−0.01	0.98 (0.95–1.01)	0.355
Multivariable	Sex, female	0.46	1.59 (0.68–3.70)	0.282
Multivariable	BMI	−0.05	0.99 (0.91–1.08)	0.907
Multivariable	eGFR	−0.02	0.97 (0.95–1.00)	0.057

Note. Dependent variable: elevated PTH level, defined as PTH > 65 pg/mL. OR, odds ratio; CI, confidence interval; BMI, body mass index; eGFR, estimated glomerular filtration rate.

**Table 4:** Sensitivity analysis using log-transformed continuous PTH as the dependent variable.

Dependent Variable	Model	Independent Variable	B	SE	β	95% CI for B	*p*
log(PTH)	Unadjusted	Uric acid/creatinine ratio	0.038	0.015	0.165	0.009 to 0.067	0.010
log(PTH)	Adjusted	Uric acid/creatinine ratio	0.033	0.014	0.143	0.005 to 0.061	0.020

Note. Linear regression analysis was performed using log-transformed PTH as the dependent variable. The adjusted model included age, sex, BMI, eGFR, corrected calcium, phosphorus, magnesium, and 25-hydroxyvitamin D. PTH: parathyroid hormone; SE: standard error; CI: confidence interval.

## Data Availability

The data that support the findings of this study are not publicly available due to patient confidentiality and ethical restrictions but are available from the corresponding author upon reasonable request.
